# The Performance of Preloaded Bolts in Seismically Prequalified Steel Joints in a Fire Scenario

**DOI:** 10.3390/ma13225079

**Published:** 2020-11-11

**Authors:** Roberto Tartaglia, Mario D’Aniello, Marco Andreini, Saverio La Mendola

**Affiliations:** 1 Department of Structures for Engineering and Architecture, University of Naples “Federico II”, Via Forno Vecchio 36, 80134 Naples, Italy; mdaniel@unina.it; 2 Occupational Health & Safety and Environmental Protection Unit, European Organization for Nuclear Research (CERN), Esplanade des Particules, 11217 Meyrin, Switzerland; marco.andreini@cern.ch (M.A.); saverio.la.mendola@cern.ch (S.L.M.)

**Keywords:** steel structures, seismic design, beam-to-column joint, bolt, fire, finite element analyses

## Abstract

Seismically pre-qualified beam-to-column joints guarantee large ductility in seismic scenarios thanks to the effectiveness of the design rules and technological requirements that are devoted to avoiding the failure of brittle components (i.e., bolts and welds). However, their performance under different severe actions like those induced by fire has not been properly investigated. Therefore, a parametric study based on finite element simulations has been carried out with the aim to verify the effectiveness of local details of seismically prequalified joints under fire. Finite element analyses were carried out on beam-to-column assemblies sub-structured from a reference archetype building accounting for both material and geometrical imperfections. The bolts’ internal actions were monitored in all the investigated specimens varying the applied vertical loads. The results show that the seismic design rules adopted to size the bolts are effective to resist the large increase in shear forces in the bolts occurring under fire. Thus, the investigated joints provide satisfactory ductility and rotation capacity at high temperature preventing the failure of bolts; further analysis could be conducted to investigated the fire performance of the investigated joints in a seismic scenario.

## 1. Introduction

Steel structures are widely used in seismic areas since they guarantee ductile and dissipative behavior by means of localized plastic deformations [1,2,3,4,5,6,7,8,9,10,11,12,13,14,15,16,17,18,19,20,21,22]. Among the wide range of structural typologies, moment resisting frames (MRFs) are the most ductile due to redundancy that guarantees plastic redistribution in plastic hinges at the beam ends. The ductility of plastic zones is highly influenced by the performance of beam-to-column joints. Therefore, the use of prequalified joints is the design strategy that guarantees reliable seismic performance [23,24,25,26,27,28,29,30,31,32,33,34,35]. In Europe, the prequalification procedure has been recently introduced following the outcomes of the EQUALJOINTS (European pre-QUALified steel JOINTS) and the EQUALJOINTS+ (European pre-QUALified steel JOINTS PLUS) research projects [36,37]. Within this framework, joints with extended stiffened bolted connections have been seismically prequalified against two different performance objectives, namely (i) elastic connection and plastic deformations developing solely into the beam end (i.e., full strength connections); (ii) plastic deformations occurring in the connection and the beam with balanced contribution (i.e., equal strength connections). Experimental tests demonstrated the effectiveness of these joints under seismic loading. However, the performance under different extreme loading conditions has not yet been properly investigated. In particular, the case of a fire scenario represents an important condition that can typically affect the structures following destructive seismic events [38]. In particular, the fire behavior of brittle components such as the bolts is a crucial issue to guarantee rotation capacity and adequate ductility under large rotation. Indeed, many Authors investigated the importance of the connections’ behavior to prevent the progressive collapse of steel buildings [39]. In particular, the bolts play a central rule to guarantee the ductility of end-plate connections when catenary action develops in the beam [40,41,42]. These considerations can be also extended to the case of seismic resistant bolted joints, which are usually designed to resist the bending moment and shear force developed by the plastic hinge into the beam, where the bolts are supposed to resist only tensile and shear forces. However, if the damage involves the connection and relative rotation occurs between the end-plate and the column flange, the bolts are subjected to bending that decreases the ultimate resistance of the bolts. This condition is even worst in case of fire owing to the degradation of the material resistance at higher temperature.

Another aspect that deserves investigation is the behavior of the joint under an increase in shear force that typically occurs due to the redistribution of forces in the frame following the increase in temperature is an aspect that has to be investigated.

These issues motivated the study summarized in this paper. Therefore, a wide range of finite element simulations was carried out with the aim to deepen the local response of the connections as well as to verify the effectiveness of local details recommended by European seismic prequalification in a fire scenario. The paper is subdivided in three main parts: the first describes the design and geometrical features of the investigated joints. In the second part the modelling assumptions and developed models are described. In the third part, the results of the parametric analyses are summarized and discussed.

## 2. Framework of the Activity and Methodology

### 2.1. Investigated Joints

A set of six extended stiffened beam-to-column joints are designed according to European seismic prequalification procedure [36,37]. In particular, both full and equal strength joints are investigated. For the sake of clarity, full strength joints are designed to concentrate plastic deformations in the connected beam, thus keeping the connection and the column web panel in elastic range. Equal strength joints are designed to develop plastic deformations within both beam and connection but keeping the column web panel free from damage. In both cases capacity design-based detailing rules are adopted to ensure ductile behavior, preventing the failure of both welds and bolts. Indeed, the connections are designed to trigger ductile failure mechanisms involving the flange yielding (i.e., failure mode 1 or mode 2 at each bolt row [34]). The beam-to-column assemblies are extracted from a set of reference moment resisting frame (MRF) properly designed in a seismic area according to the European codes [34,35]. All design data and geometrical features about these reference MRFs were reported by the Authors in a former study [43].

The buildings have a 21 × 21 m square shape with 3 spans of 7 m in both X and Y directions. The MRF are placed in the longitudinal (X) direction, while concentrical brace frames (CBF) are placed in the Y direction. The structures are designed assuming a permanent load equal to 5 kN/m^2^ and a live load equal to 3 kN/m^2^, the buildings were designed assuming a reference peak ground acceleration a_g_ equal to 0.25 g. The shape of beams and columns is a parameter of variation of the geometry of the joint. In particular, three beam–column assemblies were selected varying the steel profiles for beams and columns (see Table 1).

The parametric study in a fire scenario also included the influence of vertical loads acting on the beam. With this regard, the vertical loads applied on the beam are not evaluated according to the loading combination prescribed by the EN1990 [44], but they were systematically varied by way of the shear plastic resistance of the beam “V_pl_”. Therefore, each of the investigated assemblies was subjected to a vertical load corresponding to 0.25, 0.5 and 0.75 V_pl_.

**Figure 1:**
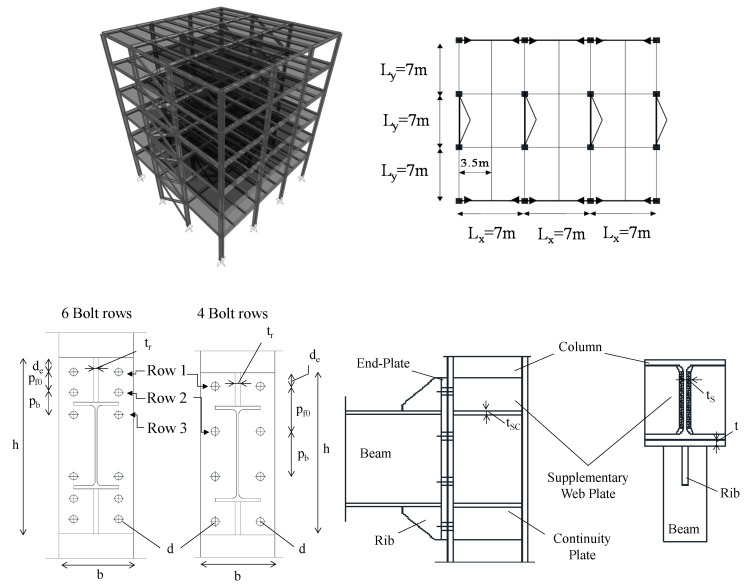
Definition of the reference structural archetype and the geometrical features of analyzed joints.

A labelling code is adopted to uniquely identify the joints by way of their geometrical features (i.e., BC1, BC2 and BC3, from those with shallow profiles to those with deep sections), the design criteria adopted (i.e., full “F” or equal “E”) and the applied vertical load (i.e., 0.25, 0.5, and 0.75 V_pl_). For instance, BC3-E-0.5V_pl_ is a bolted connection with an IPE600 as beam and HEB500 as column, designed with an equal strength connection and subjected to a vertical load equal to 50% of the beam shear resistance. The main geometrical properties of the investigated joints and the variation of the examined parameters are summarized in Table 1, while the definition of the geometrical features of the joints is described in Figure 1.

### 2.2. Finite Element Modelling Assumptions

**Figure 2:**
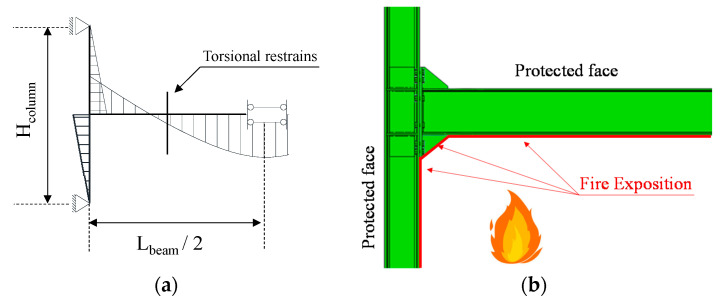
Boundary conditions (**a**) and distribution of fire on the model (**b**).

The finite element (FE) simulations were performed by means of ABAQUS 6.14 [45]. Since the joints are sub-structured from the reference MRFs, proper boundary conditions are applied to the beam-to-column assembly to simulate the restraining effect and the continuity of the surrounding parts of the MRF. Under gravity loads the moment resisting span can be reasonably assumed as fixed at both ends. Hence, the sub-structured beam–column assembly is modelled with the boundary conditions shown in Figure 2a that simplify the FE model thanks to the symmetry conditions. Torsional restrains are also applied at the beam to simulate the restraining conditions imposed by the lateral–torsional bracings (see Figure 2a) that are typically adopted in steel frames.

**Figure 3:**
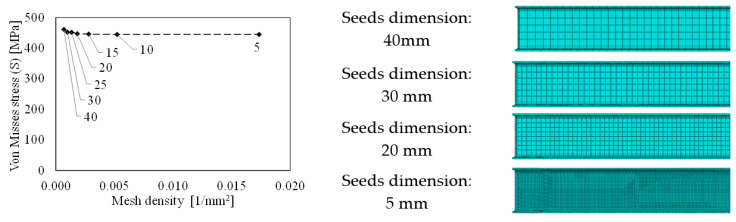
Mesh sensitivity analyses on the beam.

All parts are modelled by means of C3D8I elements (i.e., 8-node linear brick, incompatible mode) and a sensitivity analyses were performed to define the mesh dimension on each element. Figure 3 shows the variation of maximum Von Misses stresses in a predefined control point with respect to the mesh density. It can be observed that for a mesh density lager than 0.001 (that corresponds to a mesh dimension of 30 mm) the maximum stress in the control point is almost constant. Therefore, adopting an average mesh size equal to 30 mm does not influence the accuracy of the results. The same procedure was also applied to define the mesh dimension for the column, the end-plate and the bolts for which the adopted mesh dimensions are equal to 30, 10 and 5 mm, respectively.

Normal and tangential surface interactions were modelled by means of “hard contact” and “penalty” (with a friction coefficient equal to 0.4) formulations. These interactions were applied between the surfaces of the end-plate and the column flanges and the bolts (i.e., shank, head).

Tie interactions were used to model the connection between the welds and plates (e.g., the end-plate to the beam, as well as the continuity plates to column flanges).

The mill geometrical imperfections (i.e., out-of-square) of the beam flange were properly simulated by imposing a deformed shape corresponding to the buckling modes consistent with the shape of the mill imperfection. These imperfections play a central role in the characterization of the ultimate performance of full-strength joints where the plastic deformations and the ductility are highly dependent on the degradation of the plastic hinge in the beam.

Dynamic implicit quasi-static analyses were performed in three steps, namely: in the first step the tightening force was applied to the bolts by using the “Bolt load” command, where the clamping load was set equal to 321, 393 and 572 kN, respectively, for M27, M30 and M36 (in accordance with the values recommended by EN1993-1-8 [34]). Then the vertical forces were applied as uniform distribution on the upper beam flange in order to achieve a shear force in the connection alternatively equal to 0.25, 0.5 and 0.75 V_pl_. This load was applied as a uniform pressure on the top beam flange and it was kept constant during the third step which was the fire exposition. The fire was simulated as predefined field and directly applied on the elements’ surface. It is also worth noting that the fire exposed zones were not the whole surface of the assembly, but only the lower parts of the model were considered exposed to fire (see Figure 2b). This choice originates from the consideration that the fire occurs at lower floor as well as the upper parts of the beam and the connections were protected by the slab.

The temperature–time relationship was based on the nominal fire curve ISO834 according to EN 1993-1-2 (2005) [46]. As suggested by Ding and Wang [47], the temperature was considered uniform in all elements of the connection (i.e., plates, rib, bolts etc.) and its magnitude was assumed to be equal to the 80% of the bottom beam flange temperature.

**Figure 4:**
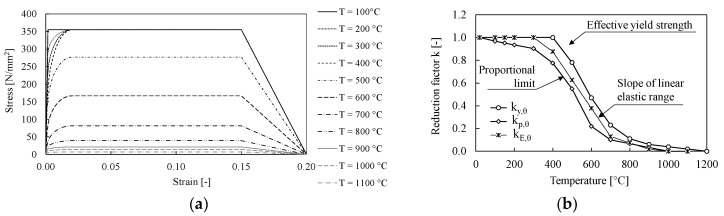
(**a**,**b**) Variation of steel mechanical properties with the temperature.

The variation of the material properties with the temperature is in line with the EN1993-1-2 [40] prescriptions. In Figure 4a,b the variations of the mechanical properties with the temperature are plotted.

S355 European steel was adopted for all the profiles and plats; the yield strength was evaluated accounting for its randomness variation and assumed equal to γ_ov_ × f_y_ =1.25 × 355 MPa = 443 MPa according to EN1998-1 [34]. The Von Misses criterion and both isotropic and kinematic hardening were accounted for in the definition of the material nonlinearity as suggested by Dutta et al. [48].

All bolts were assumed as 10.9 grade and their non-linear behavior was modelled by a multilinear force-displacement curve [43]. Moreover, according to the model proposed by Paplovic et al. [49], the ductile damage was introduced to account for both the shank necking and the fracture in the threaded part. The welds were modelled through an elastic perfectly plastic constitutive law, with the yield stress equal to 460 MPa (see Table 2).

The material thermal properties were defined according to the prescription of EN1993-1-2 [46], where the stress–strain law of steel at elevated temperatures depends on the reduction coefficient (k_y,θ_), while the k_E,θ_ factor is used to reduce the elastic modulus (E). The evolution of the both stress–strain curve and the value of the reduction coefficient with temperature are summarized in Figure 4a,b, respectively.

### 2.3. Validation of FE Models

**Figure 5:**
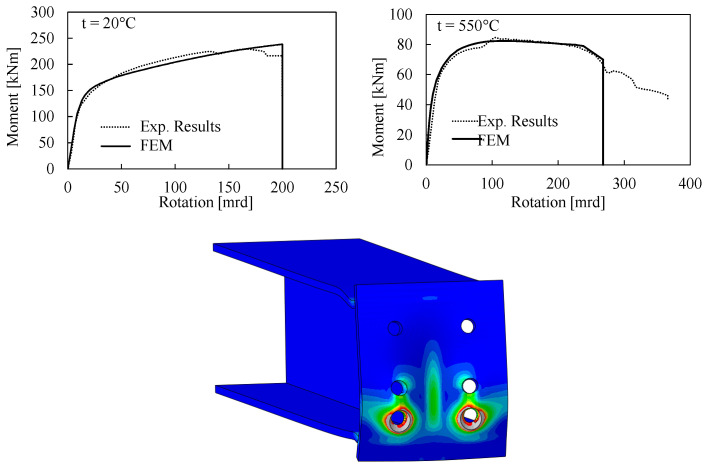
Validation of the finite element (FE) modelling procedure against the experimental results by Qiang et al. [50,51].

The accuracy of the finite element modelling assumptions was verified against the experimental results obtained by Qiang et al. [50,51]. Figure 5 depicts the comparison between the FE prediction and the experimental results in terms of moment rotation curve and equivalent plastic deformations (PEEQ). It can be observed that the adopted modelling assumptions are able to well predict the joint behavior at both ambient (20 °C) and elevated temperature (550 °C) up to a rotation of 270 mrd.

## 3. Discussion of Results

The results from finite element simulations are reported in terms of bolt forces vs. joint rotation, Von Misses stresses and equivalent plastic deformations (PEEQ).

**Figure 6:**
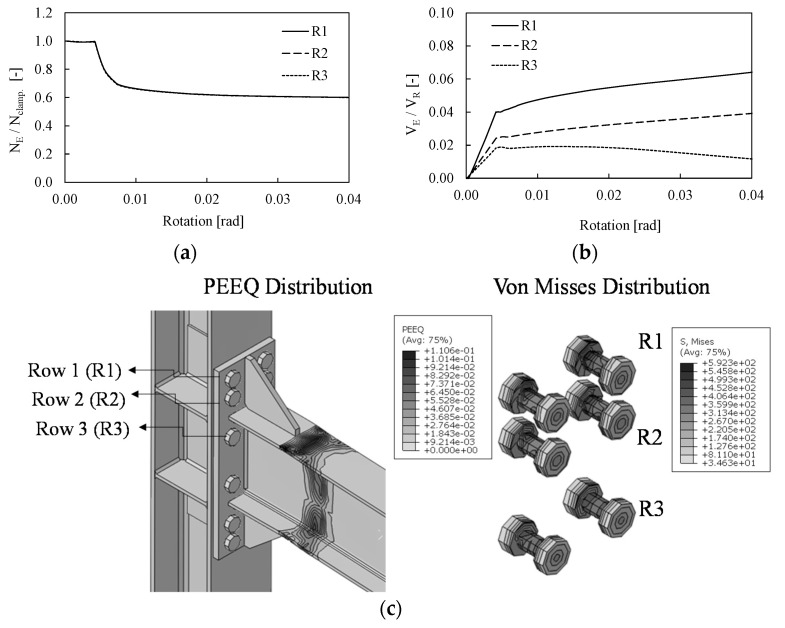
Axial (**a**) and shear (**b**) force distribution among the bolt rows in tension, plastic deformation in the joint and Von Misses stress of the bolt rows in tension of the BC1-F-0.75Vpl joint (**c**).

Figure 6 depicts the evolution of both axial and shear force developed within the bolt rows in tension (i.e., R1, R2 and R3) of BC1-F-0.75V_pl_ assembly. The axial tensile force is normalized to the applied clamping force (N_clamp_), while the shear forces are normalized to the bolt shear resistance (V_R_), where both N_clam_ and V_R_ are evaluated according to the EN1993-1-8.

For a low value of rotation, where only vertical loads are applied (see step 2), the bolt forces are constant and equal to the imposed clamping force. At this stage the connection is subjected to both shear and bending moment and the tensile forces in the bolts are almost constant and lower than the clamping action (see step 1). Contrariwise, when fire is applied (see step 3) a smooth decrease in tensile forces can be observed in all bolt rows. Indeed, as expected, due to the temperature variation the mechanical properties of steel decrease and an overall relaxation of the stress state can be observed at the initial stage. Due to the release of flexural stiffness the bending moment at the beam end deceases but the shear forces increase as a direct consequence to guarantee the system equilibrium. Figure 6b shows the evolution of the shear forces in the bolt rows with the imposed rotation. At this stage the vertical loads are applied on the beam and the distribution of shear force is almost linear in the bolts. When fire is applied with the increase in temperature the shear forces in the bolts significantly increase as a direct consequence of the overall increase in shear in the beam. For instance, with reference to the first bolt row (i.e., R1 as shown in Figure 6c) the shear force increases of about 40%, namely from 12.42 kN once the vertical load is fully applied on the beam up to 20 kN after fire is applied and the rotation reaches the value of 0.04 rad.

It is worth noting that the distribution of shear forces among the bolts is not uniform. Indeed, the first and the second rows are the most stressed, while the third row exhibits shear force lower than 38%. Figure 6c shows the distribution of PEEQ in the assembly and the Von Mises stress in the bolts where it can be observed that plastic deformations occur in the beam only and all bolts behave in elastic range.

**Figure 7:**
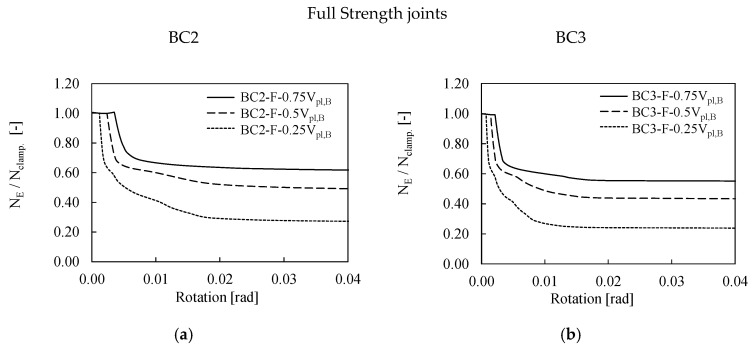

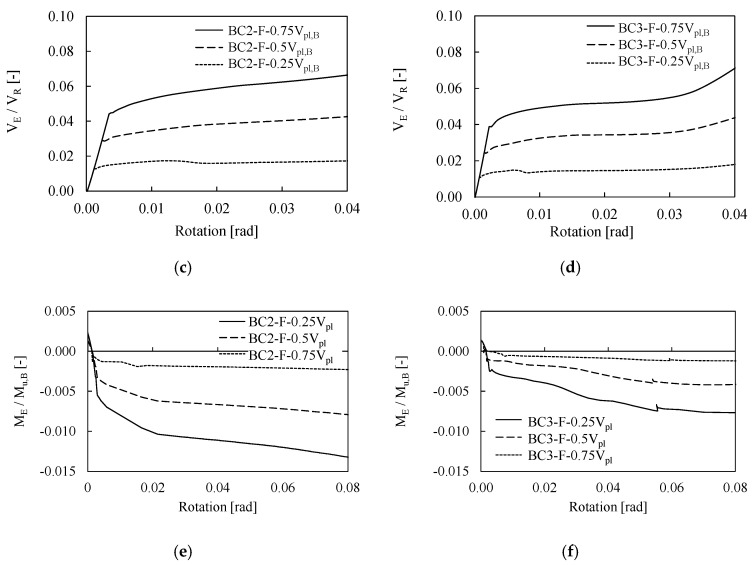
Bolt forces (**a**–**d**) and bending moments (**e**,**f**) in BC2-F and BC3-F assemblies.

**Figure 8:**
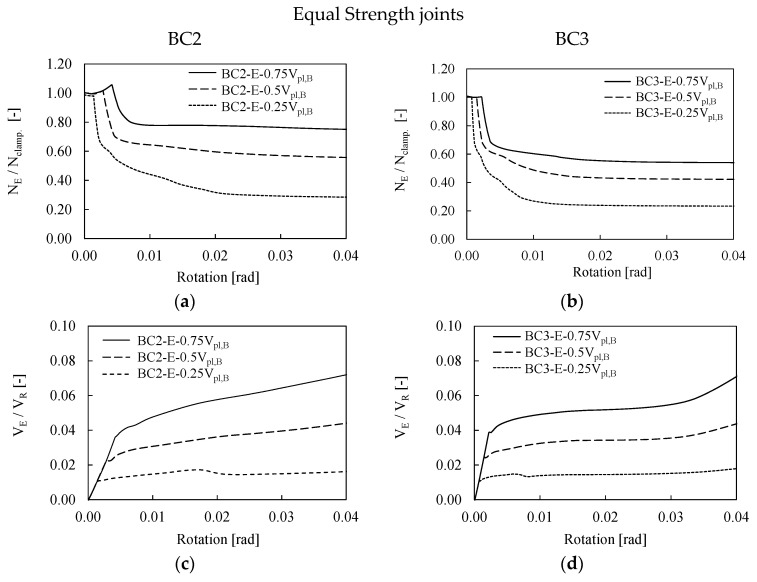

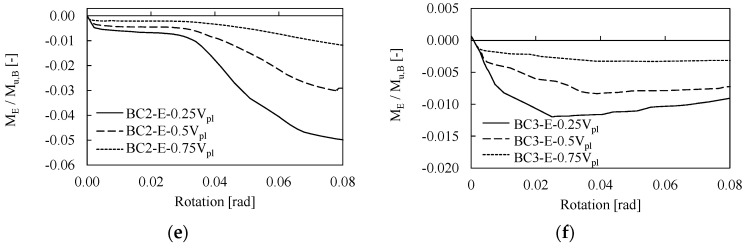
Bolt forces (**a**–**d**) and bending moments (**e**,**f**) in BC2-E and BC3-E assemblies.

Figure 7 and Figure 8 summarize the results of the first bolt row of BC2 and BC3 assemblies designed as full and equal strength, respectively. In both cases, the evolution of bolt forces is almost the same and the increase in shear forces in the bolts in a fire scenario clearly depends on the modification the rigidities of the structural system to which a variation of the distribution of internal forces is associated. However, it is important to notice that the variation of vertical loads (i.e., from the 0.25 V_pl_ to 075 V_pl_) does not significantly influence the distribution of bolt forces. The main difference is related to the threshold of rotation at which the clamping action is exceeded as shown by different widths of the horizontal plateau in Figure 7 and Figure 8. It is interesting to observe in which terms tensile forces in the bolts of the BC2-E assembly slightly exceed the clamping action in the second step (see Figure 8a). This result depends on the performance of the joint that behaves as equal strength, namely the connection is weaker and more deformable than the corresponding full strength. However, this behavior cannot be observed in the BC3-E where the distribution of tensile force in the bolts is very similar to the pattern showed by the full strength connection, as also confirmed by the PEEQ distribution (see Figure 8). The reason of the different performances of BC2-E and BC3-E depends on the different number of bolt rows, namely four and six, respectively. The greater number of bolts of BC3-E is associated to an increase in stiffness and resistance of the connection that makes the performance of BC3-E joint closer to full strength connections, as also confirmed by the smaller plastic deformation within the connection. These considerations were also confirmed by Tartaglia et al. [37] where the joint was seismically investigated.

The bending moment within the bolt rows was also monitored in order to investigate if the potential gap opening of the connection affects the resistance of the bolts.

Figure 7e,f and Figure 8e,f show the ratio between the bending moment (M_E_) acting on the bolt rows over their ultimate flexural resistance (M_u,B_) for an increasing value of applied rotation. As expected, in all investigated cases the bending moments are negligible even at large rotation greater than 4%, since the plastic deformations are mostly concentrated in the beam end. However, coherently with what was expected, the BC2-E show the largest value of the bending moment, which is about four times greater than the value developed in the corresponding full-strength configuration.

**Figure 9:**
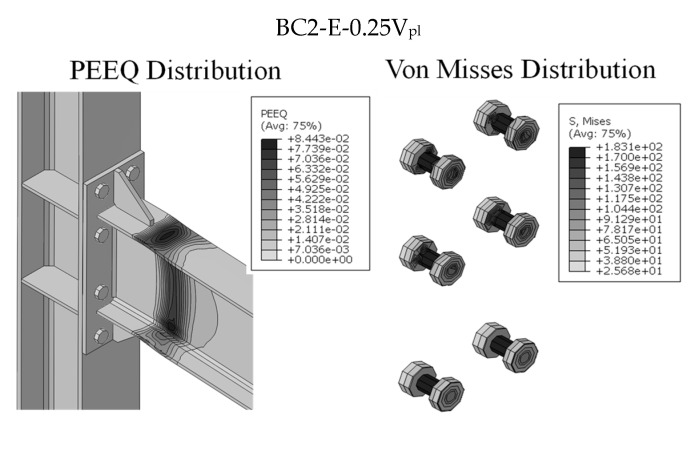

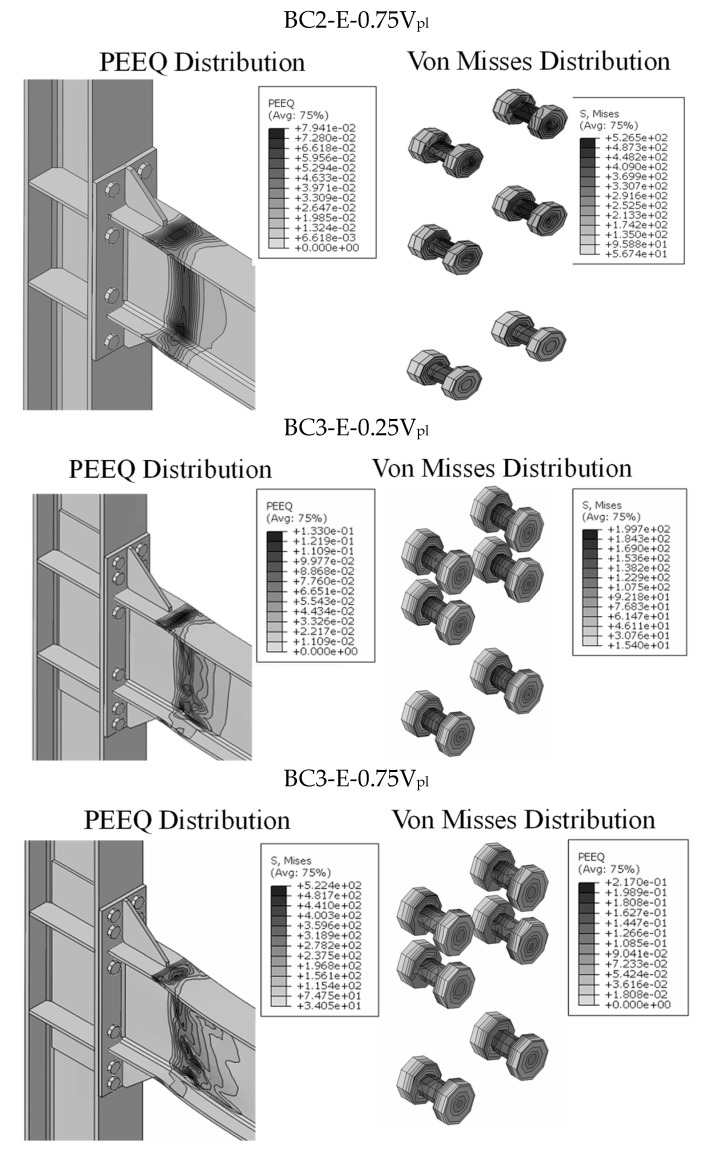
BC2 and BC3 equal strength joints: plastic deformations and Von Misses stress.

Although when increasing the vertical loads there is an increase in shear force in the bolts, the maximum V_E_/V_R_ ratio is equal to 8%; this means that the maximum stress within the bolts is less than 10% of their maximum shear capacity. These results are also confirmed by the distributions of PEEQ and Von Misses stress (see Figure 9) for both the BC2-E and the BC3-E joints subjected to a vertical load equal to 0.5 V_pl_. Indeed, as expected, all plastic deformations are concentrated in the connected beams, while all bolts behave in the elastic range. The results of the analyses shows that the increase in shear action within the bolts slightly depends on the applied vertical load because the vertical shear force is also transferred by friction between the end-plate of the connection and the column flange on the compression side.

As suggested by the EN1993-1-8 an additional verification accounting for the contemporary presence of both tensile and shear forces on the bolt rows was performed (Equation (1)) and summarized in Table 3.

(1) $$\frac{F_{v,Ed}}{F_{v,Rd}}+\frac{F_{t,Ed}}{1.4\cdot F_{t,Rd}}\leq 1$$

where *F_v,Rd_* is the bolt shear resistance defined in Equation (2) and *F_t,Rd_* is the bolt tensile resistance as defined in Equation (3):

(2) $$F_{v,Rd}=\frac{\alpha_{v}\cdot f_{u}\cdot A_{s}}{\gamma_{M2}}$$

(3) $$F_{t,Rd}=\frac{k_{2}\cdot f_{u}\cdot A_{s}}{\gamma_{M2}}$$

where according to EN1993-1-8 *k*_2_ is equal to 0.9, the *f_u_* is the ultimate bolt resistance equal to 1000 MPa in the investigated cases, *A_s_* is the effective area of the bolt, *α_v_* is function of the bolt class (equal to 0.5 for 10.9) and *γ*_*M*2_ is the partial safety factor equal to 1.25. As can be observed, all bolts comply with Equation (1).

## 4. Conclusions

A parametric study based on finite element simulations was carried out to investigate the fire behavior and the relevant local response of seismically prequalified extended stiffened bolted beam-to-column joints. On the basis of the obtained results the following remarks can made:

- Consistently with recent studies, the shear forces in the bolts significantly increase in a fire scenario, which depends on the modification the rigidities of the structural system to which a variation of the distribution of internal forces is associated.
- The fire performances of the joints depend on the applied vertical loads, thus increasing the shear action on the beam which corresponds a reduction in the joints’ capacity under fire. However, the increase in shear action within the bolts is less affected by the applied vertical load because the shear force applied on the beam is also transferred by friction between the end-plate of the connection and the column flange on the compression side.
- In all investigated cases, the plastic deformations are mostly concentrated at the beam extremities, leaving connection (i.e., the bolts and the end-plate) in elastic range.
- Despite the large increase in the shear force in the bolts, their failure was not observed in any of the investigated cases. This result confirms the effectiveness of the design seismic rules that allow to attainment of over-resistant bolts also able to resist a fire scenario.

## Figures and Tables

**Table 1:** Geometrical features and configuration of the investigated specimens.

Label	Design Performance	Shear Action	Beam Profile	Column Profile	Bolts		End-Plate		
					Rows	Diameter	H	b	t
					-	mm	mm	mm	mm
BC1-F-0.25V_pl_	Full strength	0.25 V_pl_	IPE360	HEB280	6	30	870	280	25
BC1-F-0.5V_pl5_		0.5 V_pl_							
BC1-F-0.75V_pl_		0.75 V_pl_							
BC1-E-0.25V_pl_	Equal strength	0.25 V_pl_	IPE360	HEB280	4	27	600	280	18
BC1-E-0.5V_pl5_		0.5 V_pl_							
BC1-E-0.75V_pl_		0.75 V_pl_							
BC2-F-0.25V_pl_	Full strength	0.25 V_pl_	IPE450	HEB340	6	27	1100	300	30
BC2-F-0.5V_pl5_		0.5 V_pl_							
BC2-F-0.75V_pl_		0.75 V_pl_							
BC2-E-0.25V_pl_	Equal strength	0.25 V_pl_	IPE450	HEB340	4	30	770	300	20
BC2-E-0.5V_pl5_		0.5 V_pl_							
BC2-E-0.75V_pl_		0.75 V_pl_							
BC3-F-0.25V_pl_	Full strength	0.25 V_pl_	IPE600	HEB500	6	36	1100	300	22
BC3-F-0.5V_pl5_		0.5 V_pl_							
BC3-F-0.75V_pl_		0.75 V_pl_							
BC3-E-0.25V_pl_	Equal strength	0.25 V_pl_	IPE600	HEB500	6	36	1100	300	18
BC3-E-0.5V_pl5_		0.5 V_pl_							
BC3-E-0.75V_pl_		0.75 V_pl_							

**Table 2:** Material properties.

Elements	Material	Elastic Modulus	Yield Stress	Ultimate Stress
		N/mm^2^	N/mm^2^	N/mm^2^
Bolts	10.9	210,000	900	1000
Profiles and Plates	S355	210,000	355	510
Welds	S460	210,000	460	460

**Table 3:** Verification of bolts under combined shear and tensile forces.

Specimens	Configuration	Bolt Resistance (Capacity “C”)			Bolts Forces (Demand “D”)		Verification	
		Bolt Rows	N_Rd_^1^	V_Rd_^2^	N_Ed_^3^	V_Ed_^4^	D/C	C/D
		-	kN	kN	kN	kN	-	-
BC1-F	0.25 V_pl_	6	3054	1031	2358	11.56	0.56	1.78
	0.5 V_pl_	6	3054	1031	2358	31.41	0.58	1.72
	0.75 V_pl_	6	3054	1031	2358	38.37	0.59	1.70
BC1-E	0.25 V_pl_	4	1649	687	1927	10.92	0.85	1.18
	0.5 V_pl_	4	1649	687	1944	20.72	0.87	1.15
	0.75 V_pl_	4	1649	687	1974	26.3	0.89	1.12
BC2-F	0.25 V_pl_	6	3054	1031	2358	13.21	0.56	1.77
	0.5 V_pl_	6	3054	1031	2358	14.79	0.57	1.77
	0.75 V_pl_	6	3054	1031	2360	48.73	0.60	1.67
BC2-E	0.25 V_pl_	4	2036	848	2360	8.91	0.84	1.19
	0.5 V_pl_	4	2036	848	2370	18.7	0.85	1.17
	0.75 V_pl_	4	2036	848	2418	27.75	0.88	1.13
BC3-F	0.25 V_pl_	6	4397	1832	3432	23.7	0.57	1.75
	0.5 V_pl_	6	4397	1832	3432	62.7	0.59	1.69
	0.75 V_pl_	6	4397	1832	3432	106.3	0.62	1.62
BC3-E	0.25 V_pl_	6	4397	1832	3432	16.02	0.57	1.77
	0.5 V_pl_	6	4397	1832	3432	44.79	0.58	1.72
	0.75 V_pl_	6	4397	1832	3432	77.6	0.60	1.67

^1^ Bolt axial capacity; ^2^ Bolt shear capacity; ^3^ Bolt axial demand; ^4^ Bolt shear demand.
